# Expression and purification of LipL32 recombinant protein of *Leptospira *interrogans in different *E.coli *strains

**DOI:** 10.1186/1753-6561-8-S4-P165

**Published:** 2014-10-01

**Authors:** Victor Bastos, Jonny Yokosawa, Paulo Ho

**Affiliations:** 1 Universidade Federal de Uberlândia, UFU, Santa Monica, Uberlândia, MG, Brazil; 2 Instituto de Ciências Biomédicas, Universidade Federal de Uberlândia, UFU, Santa Monica, Uberlândia, MG, Brazil; 3 Centro de Biotecnologia, Instituto Butantan, São Paulo, SP, Brazil

## Background

Leptospirosis is a zoonosis caused by the infection of pathogenic bacteria of the genera Leptospira, often transmitted by direct or indirect contact with urine of infected animal hosts [1]. In national territory, leptospirosis is considered an endemic disease, with high incidence in raining seasons, but occurring during the whole year [2]. From all the cases, 15% evolve to the icteric form, with serious clinic manifestations, as severe icteric, hemorrhages and kidney disease, including lethal onset in 50% of the cases [3].

From all membrane proteins LipL32 is the most abundant and found in all pathogenic species of Leptospira. Saprophytic strains do not express LipL32, making the detection of the protein or the detection of specific antibody a potential toll for the development of a laboratorial diagnosis [4].

## Methods

DNA of pAE-lipL32 was utilized in the transformation of E. coli strains BL21-S1, with expression induced by NaCl, and BL21 Star(DE3)pLysS, with expression induced by IPTG. Eight transformed colonies of each strain were inoculated in culture medium, and after the induced expression and analysis, by SDS-PAGE, the results showed a high level of expression in BL21-SI strains. One of the BL21-SI clone was chosen to express the LipL32 protein in a higher volume, for purification in a anionic chromatographic column, using a crescent concentration of ambic elution buffer (0,05 to 1,0 M). The purification resulted in 295 fractions combined in 15 proteic pools according with the protein spikes observed by spectrophotometry. The 15 proteic pool were analyzed in SDS-PAGE.

## Results and conclusions

The SDS-PAGE showed only one band corresponding to the LipL32 protein, from pools six to 15, although, pool six has a visible higher expression, and pools 14 and 15 showed the presence of contaminant proteins, probably due to the high concentration of the eluent. More purifications steps are needed, but the expression of LipL32 by E. coli strain BL21-SI, induced by NaCl, was successfully achieved.
